# Development of a machine learning-based model for predicting positive margins in high-grade squamous intraepithelial lesion (HSIL) treatment by Cold Knife Conization(CKC): a single-center retrospective study

**DOI:** 10.1186/s12905-024-03180-2

**Published:** 2024-06-07

**Authors:** Lin Zhang, Yahong Zheng, Lingyu Lei, Xufeng Zhang, Jing Yang, Yong Zeng, Keming Chen

**Affiliations:** Department of Obstetrics and Gynecology, The First Affiliated Hospital of Yangtze University, Shashi District, 8 Hangkong Road, Jingzhou, Hubei China

**Keywords:** HSIL, CKC, Positive margin, Predictive model, Machine learning

## Abstract

**Objectives:**

This study aims to analyze factors associated with positive surgical margins following cold knife conization (CKC) in patients with cervical high-grade squamous intraepithelial lesion (HSIL) and to develop a machine-learning-based risk prediction model.

**Method:**

We conducted a retrospective analysis of 3,343 patients who underwent CKC for HSIL at our institution. Logistic regression was employed to examine the relationship between demographic and pathological characteristics and the occurrence of positive surgical margins. Various machine learning methods were then applied to construct and evaluate the performance of the risk prediction model.

**Results:**

The overall rate of positive surgical margins was 12.9%. Independent risk factors identified included glandular involvement (OR = 1.716, 95% CI: 1.345–2.189), transformation zone III (OR = 2.838, 95% CI: 2.258–3.568), HPV16/18 infection (OR = 2.863, 95% CI: 2.247–3.648), multiple HR-HPV infections (OR = 1.930, 95% CI: 1.537–2.425), TCT ≥ ASC-H (OR = 3.251, 95% CI: 2.584–4.091), and lesions covering ≥ 3 quadrants (OR = 3.264, 95% CI: 2.593–4.110). Logistic regression demonstrated the best prediction performance, with an accuracy of 74.7%, sensitivity of 76.7%, specificity of 74.4%, and AUC of 0.826.

**Conclusion:**

Independent risk factors for positive margins after CKC include HPV16/18 infection, multiple HR-HPV infections, glandular involvement, extensive lesion coverage, high TCT grades, and involvement of transformation zone III. The logistic regression model provides a robust and clinically valuable tool for predicting the risk of positive margins, guiding clinical decisions and patient management post-CKC.

## Introduction

Cervical high-grade squamous intraepithelial lesion (HSIL), which encompasses cervical squamous intraepithelial neoplasia grades 2 (CIN2) and 3 (CIN3), is closely associated with the development of invasive cervical cancer. Studies suggest that if left untreated, approximately 5% of CIN2 lesions and 12% to 33% of CIN3 lesions may progress to invasive cancer, reflecting significant variability based on lesion severity [1]. Moreover, recent literature, such as McCredie [2], notes that the cumulative incidence of invasive cancer over 30 years can reach 31.3% in women with substantial colposcopically visualized CIN3 managed only by biopsy, with a higher incidence in those with persistent disease. Of course, the current risk of progressing to invasive cancer is low the reported in previous, possibly as earlier and more sensitive detection methods like liquid-based cytology and HPV testing are likely to identify lesions at a stage where they pose a lower risk of progression. Given the potential for progression, particularly in untreated CIN3, and the significantly reduced risk of 0.7% following conventional surgical treatment [1], proactive surgical management of HSIL is recommended to prevent the development of cervical cancer. Cervical conization is an important method for the diagnosis and treatment of cervical precancerous lesions. The specimen can be retained for histological evaluation and the margin status of the lesion can be determined. However, cervical conization is different from traditional gynecological surgery. There is no enveloped-like structure of the cervix, no texture abnormality can be reached, the lesion on the surface of the cervix can be seen, and the lesion in the cervical canal cannot be defined. Therefore, even if the standardized operation is strictly followed, the positive margin of the conization sample cannot be completely avoided [3].A retrospective study by Zeng found that positive resection margin was an independent risk factor for residual lesions after HSIL conization [4]. Another study found that compared with patients with negative resection margin, the relative risk of persistent/recurrent HSIL in patients with positive resection margin after one year of treatment was 11.36 times greater (95%CI: 5.529–23.379, *P* < 0.0001)[5]. However, there is no unified conclusion on the related risk factors of positive resection margin after conization. The aim of this study is to analyze the risk factors of positive surgical margin after cold knife conization (CKC) in patients with HSIL in our hospital, and to establish a predictive model to provide guidance for individualized management of HSIL patients after conization.

## Materials and methods

**Fig. 1:**
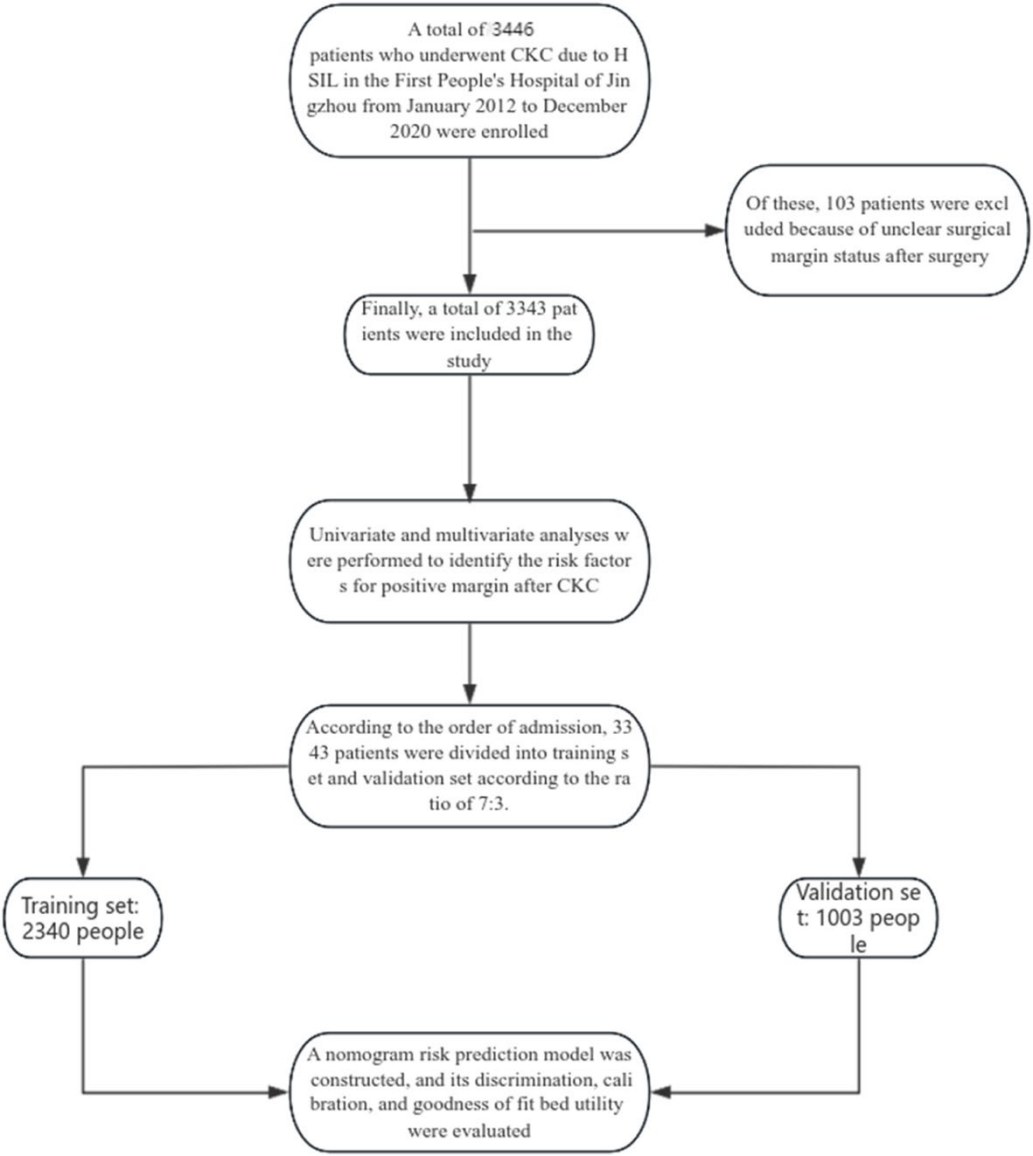
Flow chart

The clinical data of patients who underwent CKC for HSIL in the First Affiliated Hospital of Yangtze University from January 2012 to December 2022 were collected.The study was approved by the Ethics Committee of the First Affiliated Hospital of Yangtze University. Informed consent was obtained from all patients or their families. The following criteria:①Colposcopy was performed before operation and the cervical pathological biopsy was HSIL; ②The initial treatment was CKC; ③ The pathological examination results after cervical conization were still HSIL; ④No previous history of HSIL diagnosis and surgical treatment; ⑤Complete clinicopathological data were available. According to the order of admission, the enrolled patients were divided into training set and validation set according to the ratio of 7:3. (Fig. 1 Flow chart).

For human papillomavirus (HPV) testing and genotyping, the Cobas HPV test (Cobas 4800; Roche Molecular Diagnostics), based on a real-time polymerase chain reaction (PCR) system. The assay detects 18 high-risk HPV (HR-HPV) types and provides specific information on HPV 16/18 infection. Multiple HR-HPV infections were defined as two or more HR-HPV infections.

Membrane-thin layer liquid-based cytology: Epithelial cells of the cervix were collected by cervical brush and diagnosed by a cytologist according to the TBS (2001) system (Xiaogan Aohua Medical Technology Limited Liability Company). Examination results: No abnormal cells (NILM), atypical squamous cells of unknown significance (ASCUS), low-grade squamous intraepithelial lesion (LSIL), atypical squamous cells of cannot exclude high-grade squamous intraepithelial lesion (ASC-H), high-grade squamous intraepithelial lesion (HSIL).Low-level lesion: ASCUS, LSIL; High-level lesion: ASC-H, HSIL.

The cervical intraepithelial lesion was divided into HSIL and LSIL according to the cervical lesion nomenclature standard issued by the American Society for Pathology (CAP) and American Society for Colposcopy and Cervical Pathology (ASCCP) in 2012. HSIL includes CIN 2 and 3 [6]. Patients with cervical intraepithelial lesions were reclassified according to this protocol.

Cervical Transformation Zone (TZ): according to the international terminology of colposcopy, the types of cervical transformation zone are divided into three types: Type I: the cervical transformation zone is completely located outside the cervical canal and can be fully displayed. Type II: part of the cervical transformation zone is located outside the cervical canal, but the part of the cervical canal can still be displayed completely. Type III: part of the transformation area within the cervical canal is not fully visible.

Positive margin: If cervical intraepithelial neoplasia 1–3 was found approximately 1 mm or below the margin of resection, the margin status of the conized specimen was considered positive. Including: endocervical resection margin, ectocervical resection margin and combined resection margin. A copositive margin means that both the endocervical and ectocervical margins are positive.

Lesion covering: The cervix was divided into four quadrants and the extent of the lesion was determined based on the number of affected quadrant.

## Statistical analysis

SPSS (version 26.0), R (version 4.3.1) and python were used for data analysis. The SPSS software was used to find out the high risk factors of CKC positive surgical margin by univariate and multivariate analysis, and draw a nomogram according to these factors. The total data set was divided into two parts according to the order of admission, of which 70% constituted the training set and 30% constituted the validation set. A five-fold cross-validation scheme was used for testing. The Machine learning models tested included Logistic Regression (LR), Support Vector Machine (SVM), K-Nearest Neighbor (KNN), DecisionTree, RandomForest, eXtreme Gradient Boosting (XGBoost) and NaiveBayes were used in this study. The constructed model was used to predict the individuals in the training set and the validation set respectively. The accuracy, sensitivity, specificity, positive predict value (PPV), negative predict value (NPV) and area under curve (AUC) of the receiver operating characteristic curve were used to evaluate the predictive performance of the model. Internal and external validation results were used to determine the optimal prediction model for positive margin after CKC. The confusion matrix was used to compare the prediction results of the model with the true category of the sample. Sensitivity analysis was used to discuss the steady state of the prediction model. The predictive value was evaluated according to the area under the receiver operating characteristic (ROC) curve calculated by the regression model. Identify cut-off based on the sum of sensitivity and specificity. AUC ranged from 0.9 to 1, indicating a high predictive value; AUC ranged from 0.7 to 0.9, indicating a good predictive value; AUC ranged from 0.5 to 0.7, indicating an average predictive value; AUC < 0.5, indicating no predictive value. The calibration curve was used to evaluate the calibration of the established model. Hosmer–Lemeshow goodness of fit test and calibration curve were used to evaluate the goodness of fit and calibration of the established model. Decision curve analysis (DCA) was used to evaluate the clinical application value of the model. The model was validated by internal validation (Bootstrap self-sampling 1000 times method) and external validation (validation set). *p* < 0.05 was considered statistically significant.

## Results

A total of 3343 patients were included in the study. Among them, 432 patients had positive surgical margins, the total positive rate of surgical margins was 12.9%. including 191 cases (44.2%) endocervical resection positive margin, 130 cases (30.1%) ectocervical resection positive margin, and 111 cases (25.7%) of combined positive surgical margins. The average age of the patients was 46.7 years, and the median age was 47 years (range 17–84 years). There were 1339 patients (40.1%) aged ≥ 50 years. There were 1273 postmenopausal patients (38.1%). There were 2955 cases (88.4%) of HR-HPV infection, including 1725 cases (51.6%) of HPV16/18 infection, and 1199 cases (35.9%) of multiple high-risk HPV infection. The TCT showed: 879 cases NILM, 1009 cases ASC-US, 706 cases LSIL, 382 cases ASC-H, 367 cases HSIL, the positive rate of surgical margin was 7.5%, 7.4%, 12.5%, 53.1% and 21.3%, respectively. There were 1315 cases (39.3%) with lesions more than 3 quadrants, and the positive margin rate was 22.2%. The number of patients with TZ I/II/IIIwas 1450, 1261, and 832, respectively, and the positive margin rates were 7.7%, 8.2%, and 26% respectively.

Correlation analysis of demographic and clinicopathological parameters with positive surgical margins after CKC. Correlation analysis of demographic and clinicopathological parameters with positive surgical margins after CKC. Univariate analysis showed that: Glandular involvement (*P* < 0.001), TZ III (P < 0.001), gravidity ≥ 3 times (*P* = 0.041), parity ≥ 2 times (*P* = 0.002), 16/18 HPV infection (*P* < 0.001), multiple HR-HPV infection (P < 0.001), TCT ≥ ASC-H (P < 0.001) and Lesion covering ≥ 3 quadrants (*P* < 0.001) were associated with positive surgical margins. Age ≥ 50 (*P* = 0.346), menopause (*P* = 0.124), smoking (*P* = 0.360) and immune system diseases (*P* = 0.155) were not significantly associated with positive surgical margin. (Table 1).

**Table 1:** Univariate analysis associated with positive resection margins after CKC

Parameter	Numbers		OR (95%CI)	*P*-values
	Total	postive margin(%)		
Age(years)			1.104 (0.899–1.355)	0.346
< 50	2004	250 (12.5%)		
≥ 50	1339	182 (13.6%)		
Glandular involvement			2.167 (1.736–2.706)	< 0.001
No	1462	122 (8.3%)		
Yes	1881	310 (16.5%)		
Menopause			1.175 (0.957–1.443)	0.124
No	2070	253 (12.2%)		
Yes	1273	179 (14.1%)		
TZ types			3.726 (3.024–4.589)	< 0.001
TZ I/II	2511	216 (8.6%)		
TZ III	832	216 (26%)		
Smoke			0.768 (0.436–1.352)	0.360
No	3288	428 (11.2%)		
Yes	55	4 (7.3%)		
Gravidity			1.257 (1.009–1.565)	0.041
< 3	1152	130 (11.3%)		
≥ 3	2191	302 (13.8%)		
Parity			1.389 (1.134–1.701)	0.002
< 2	1763	197 (11.2%)		
≥ 2	1580	235 (14.9%)		
HPV-16/18 infection			2.756 (2.206–3.444)	< 0.001
No	1618	120 (7.4%)		
Yes	1725	312 (18%)		
Multiple HR-HPV infection			1.898 (1.548–2.326)	< 0.001
No	2144	219 (10.2%)		
Yes	1199	213 (17.8%)		
TCT ≥ ASC-H			3.840 (3.111–4.740)	< 0.001
No	2594	229 (8.8%)		
Yes	749	203 (27.1%)		
Immunological diseases			0.794 (0.578–1.091)	0.155
No	2899	384(13.2%)		
Yes	444	48 (10.9%)		
Lesion covering(quadrant)			3.849 (3.103–4.774)	< 0.001
< 3	2028	140 (6.9%)		
≥ 3	1315	292 (22.2%)		

The feasible variables associated with positive surgical margin found in univariate analysis were included in the logistic multivariate regression equation for multivariate analysis. Multivariate analysis showed that Glandular involvement (OR = 1.716, 95%CI: 1.345–2.189), TZ III(OR = 2.838, 95%CI: 2.258–3.568), 16/18 HPV infection (OR = 2.863, 95%CI: 2.863) 2.247–3.648), multiple HR-HPV infection (OR = 1.930, 95%CI: 1.537–2.425), TCT ≥ ASC-H (OR = 3.251, 95%CI: 2.584–4.091), Lesion covering ≥ 3 quadrants (OR = 3.264, 95%CI: 2.593–4.110) were significantly associated with positive surgical margins. (Table 2).

**Table 2:** Multivariate analysis associated with the presence of positive margins after CKC

	**B**	**S.E**	**Wald**	**Sig**	**OR(95%CI)**		
**Glandular involvement**	0.540	0.124	18.903	** < 0.001**	1.716	1.345	2.189
**TZ III**	1.043	0.117	79.898	** < 0.001**	2.838	2.258	3.568
**HPV-16/18 infection**	1.052	0.124	72.394	** < 0.001**	2.863	2.247	3.648
**Multiple HR-HPV infection**	0.658	0.116	31.945	** < 0.001**	1.93	1.537	2.425
**TCT ≥ ASC-H**	1.179	0.117	101.098	** < 0.001**	3.251	2.584	4.091
**Lesion covering ≥ 3**	1.183	0.118	101.275	** < 0.001**	3.264	2.593	4.11
Gravidity ≥ 3	0.079	0.127	0.385	**0.535**	1.082	0.843	1.389
Parity ≥ 2	-0.119	0.118	1.007	**0.316**	0.888	0.704	0.120

**Fig. 2:**
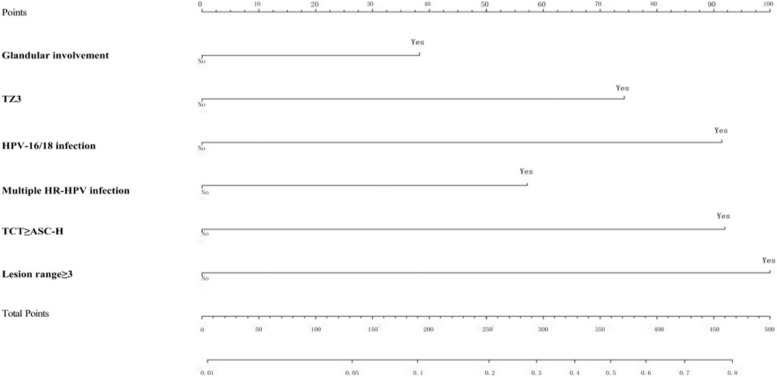
Nomogram model for the presence of positive margins after CKC

The 6 independent risk factors screened in the multivariate logistic regression analysis of the training set were used as the final predictors for the construction of the nomogram model. As can be seen from the nomogram, the scores corresponding to each predictor were summed to obtain the total score, and then, based on the predicted value corresponding to the total score, the probability of a positive predicted margin was obtained (Fig. 2).

**Fig. 3:**
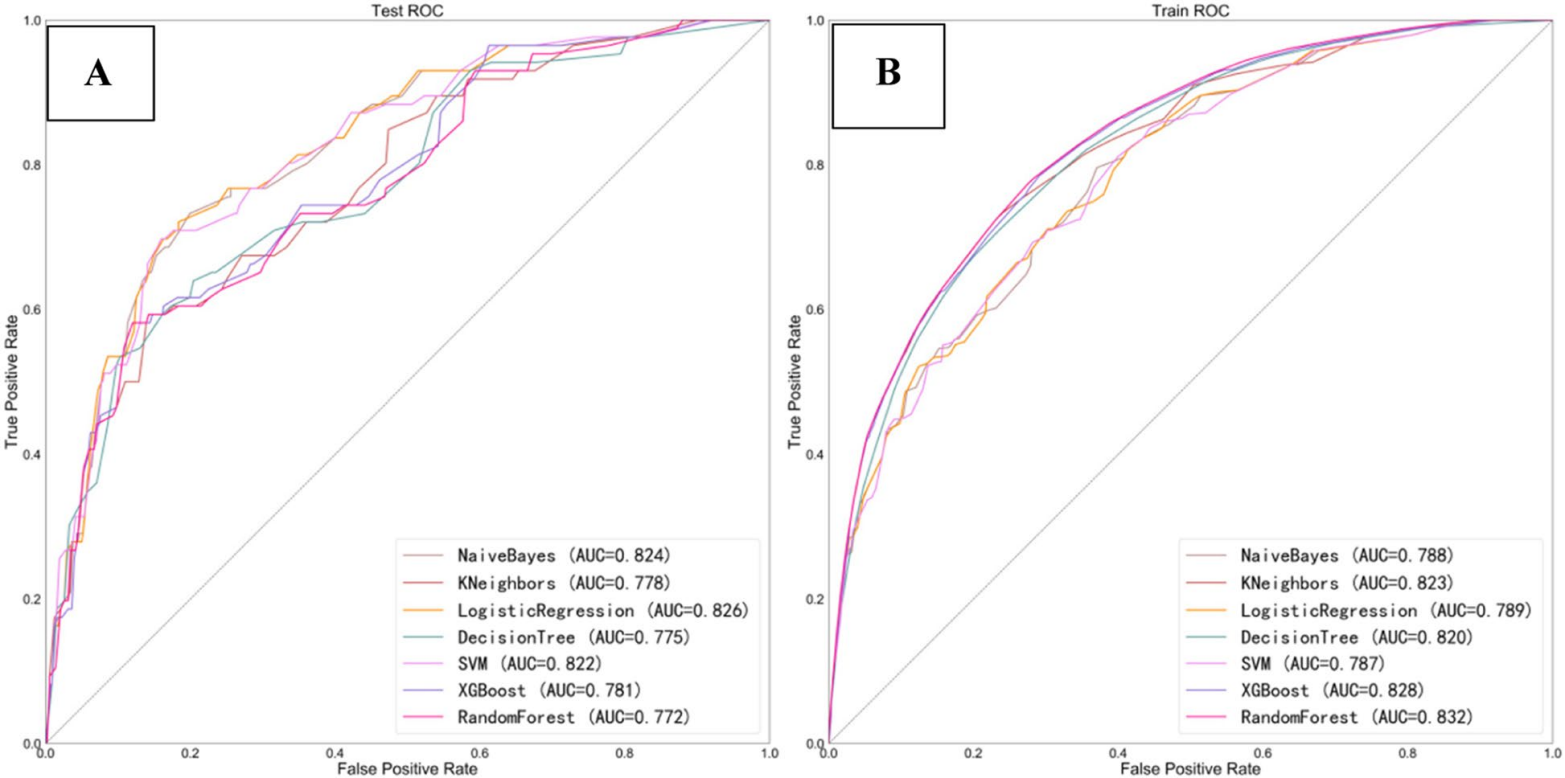
AUC curves of different machine learning prediction models in training sets (**3A**) and validation sets (**3B**)

The prediction Accuracy (ACC), Sensitivity, Specificity, Positive Predict Value (PPV), Negative Predict Value (NPV) and AUC values of the seven models in the internal training set and the external validation set are summarized. In the training set, the prediction accuracy, sensitivity, specificity, AUC and other indicators of these seven models have their advantages and disadvantages, but in the external verification results, the LR model takes into account the prediction accuracy, sensitivity and specificity, and the AUC value is the highest. Therefore, based on the results of internal testing and external verification, LR is the optimal prediction model in this study (Tables 3 and 4, Fig. 3A and B).

**Table 3:** Predictive performance of different machine learning models in training sets

Model	ACC	AUC	Sensitivity	Specificity	PPV	NPV
LogisticRegression	70.1%	0.789	0.6829	0.7195	0.7088	0.6941
NaiveBayes	70.5%	0.7884	0.7113	0.6975	0.7016	0.7073
SVM	70.2%	0.7872	0.6993	0.7036	0.7023	0.7005
KNN	74.7%	0.8225	0.7259	0.7676	0.7575	0.7369
DecisionTree	73.7%	0.8195	0.707	0.7671	0.7522	0.7236
RandomForest	74.9%	0.8318	0.7701	0.728	0.739	0.7601
XGBoost	74.7%	0.8284	0.7878	0.7053	0.7277	0.7687

**Table 4:** Predictive performance of different machine learning models in validation sets

Model	ACC	AUC	sensitivity	specificity	PPV	NPV
LogisticRegression	74.7%	0.826	0.7674	0.7444	0.3069	0.9559
NaiveBayes	72.1%	0.824	0.7674	0.7135	0.2832	0.9541
SVM	72.4%	0.822	0.7674	0.7169	0.2857	0.9543
KNN	74%	0.7784	0.6279	0.7564	0.2755	0.9323
DecisionTree	75.5%	0.775	0.6511	0.7701	0.2947	0.9373
RandomForest	69.7%	0.7719	0.6511	0.7032	0.2445	0.9318
XGBoost	69.4%	0.7807	0.6744	0.6963	0.2468	0.9354

**Fig. 4:**
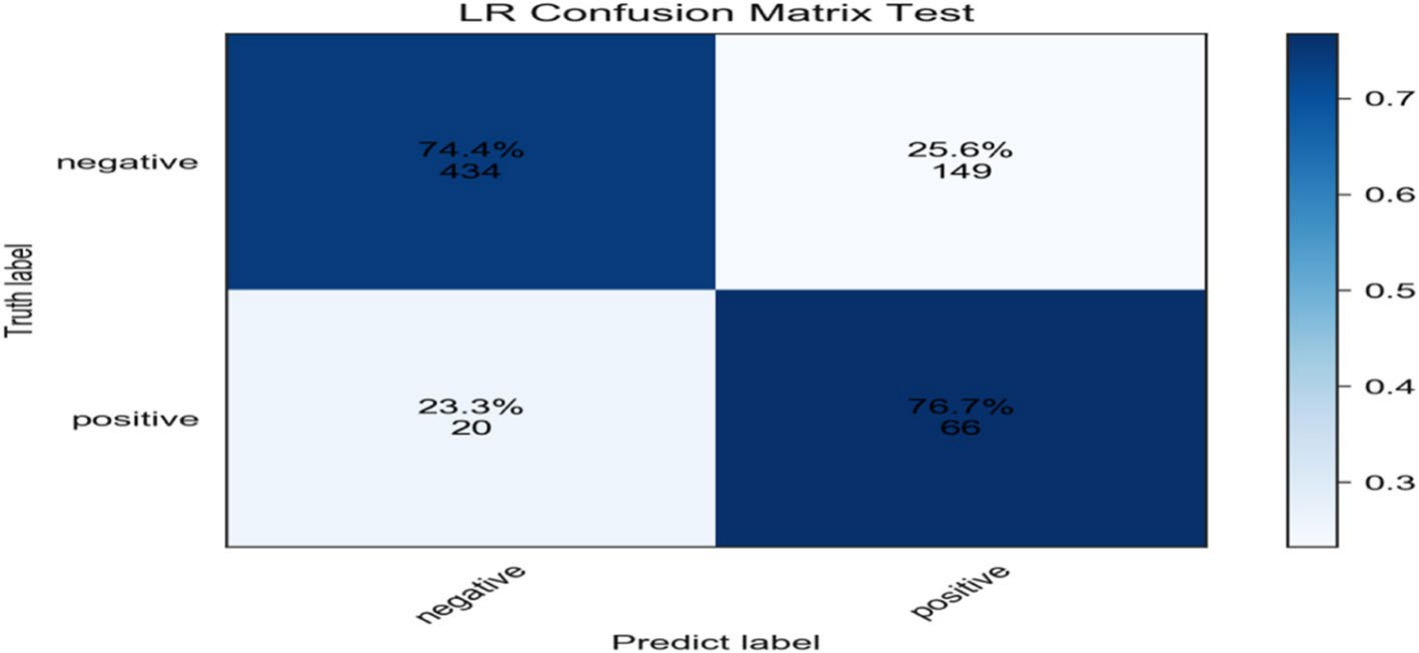
Confusion matrix of LR model on verification set

A confusion matrix was constructed based on the real and predicted values in the validation set. The prediction performance of the LR model was the best among the 7 models, and the prediction accuracy, sensitivity, specificity, PPV and NPV of the model were calculated from the matrix. The accuracy, sensitivity, specificity, PPV and NPV were 74.7%, 76.7%, 74.4%, 30.7% and 95.6% respectively in the validation set. (Fig. 4).

**Fig. 5:**
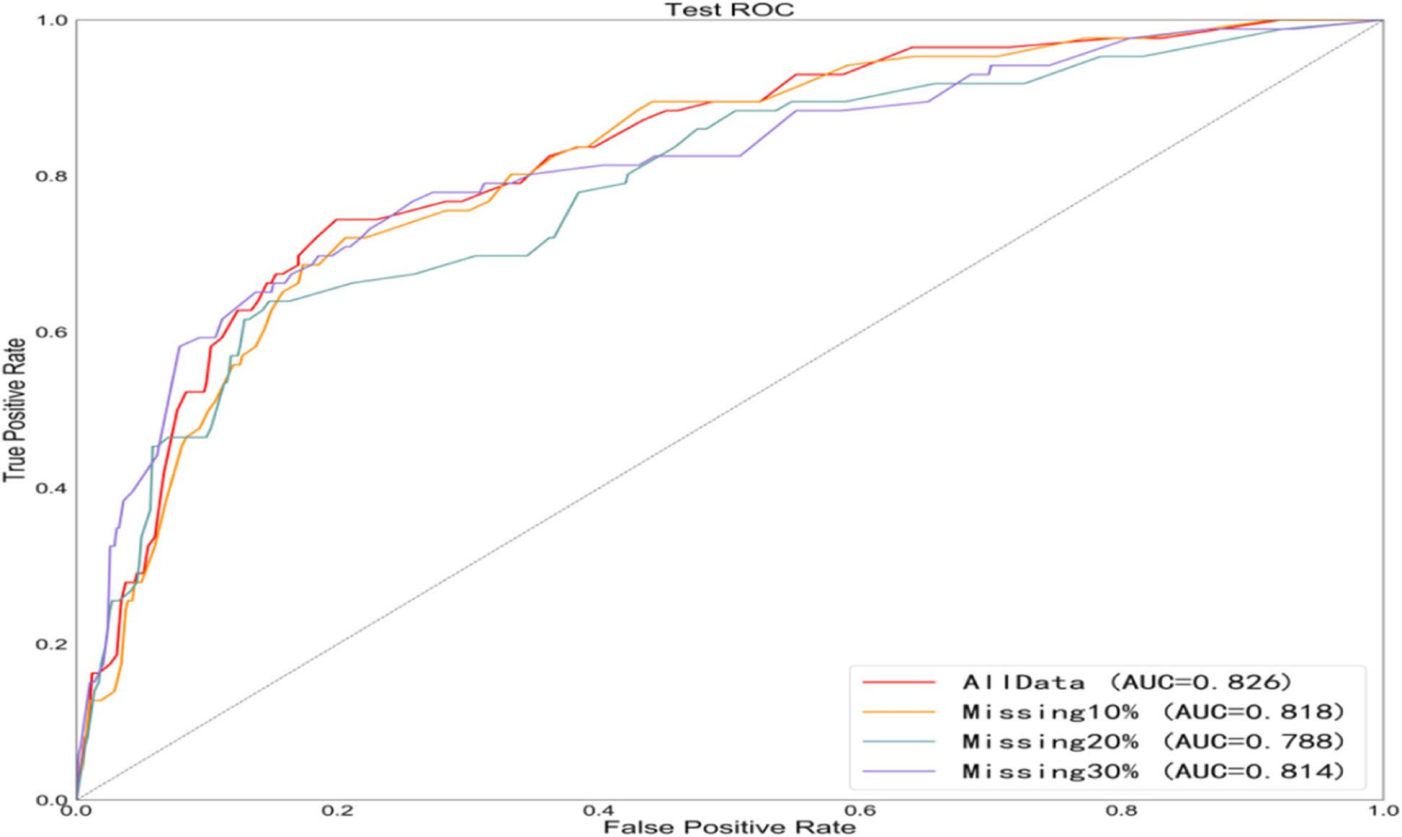
ROC curve on the validation set is obtained by creating random masks with missing values (10%, 20%, 30%)

In the validation set, we performed sensitivity analysis by centrally creating random masks with missing values (10%, 20%, 30%). The area under ROC curve obtained was all greater than 0.7, which was basically consistent with the text results. (Fig. 5).

**Fig. 6:**
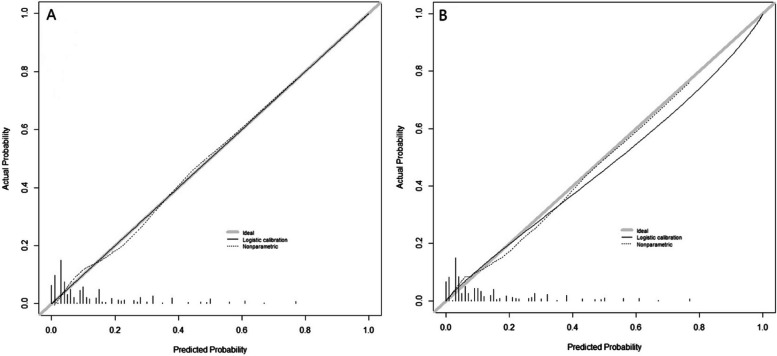
Nomogram calibration curve evaluation and validation (6A training set, 6B validation set)

The Hosmer–Lemeshow goodness of fit test and calibration curve were used to evaluate the goodness of fit and calibration of the model. Hosmer–Lemeshow test: χ2 = 10.413, *P* = 0.318 in training set, χ2 = 10.494, *P* = 0.311 in validation set, all *P* > 0.05, indicating that the model fitted well. The calibration curve in the training set showed that the shape of the predicted curve was basically consistent with the ideal curve, indicating that the risk of positive surgical margins after CKC predicted by the model was consistent with the actual risk of positive surgical margins, and the model had high accuracy. Compared with the training set, the calibration curves of the validation set were basically consistent. (Fig. 6 A and B).

**Fig. 7:**
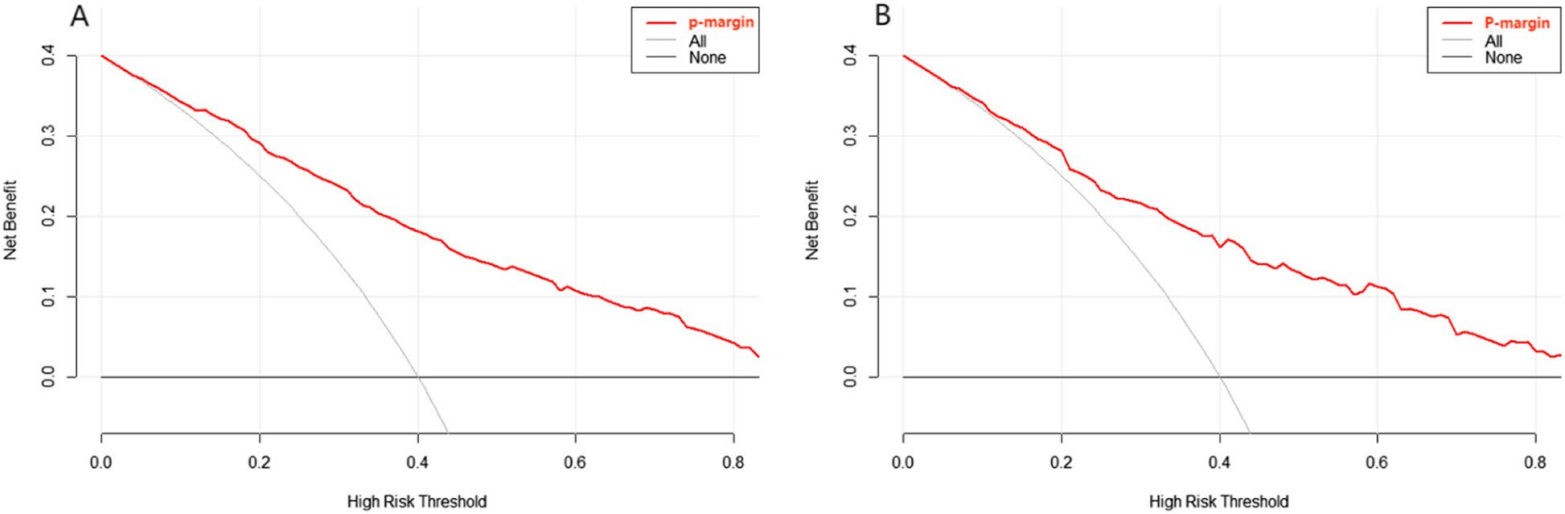
Nomogram clinical decision curve evaluation and validation (7A training set, 7B validation set)

A clinical decision curve was constructed to evaluate the clinical practicability of the prediction model. The figure in the results of this study shows that within a large threshold probability range, the red line is located at the upper right of the All line and the None line, indicating that the nomogram prediction model we constructed for positive resection margin after CKC has high clinical practical value. (Fig. 7A and B).

## Discussion

Currently, cervical conization serves as a pivotal treatment strategy for HSIL. A significant challenge in this approach is the incidence of positive resection margins, which substantially influence the risk of residual disease and recurrence of HSIL. It has been reported that the rate of positive resection margins following HSIL conization is 12.7% [7]. Furthermore, according to a meta-analysis by Arbyn [8], while the overall risk of residual or recurrent CIN2 + post-treatment is 6.6%, the occurrence of positive margins was observed in 23.1% of cases. Importantly, the risk of recurrence for those with positive margins is significantly higher at 16.9%, compared to only 3.5% for those with negative margins, indicating a relative risk of 4.8. This data highlights the critical need for precision in surgical technique to ensure complete removal of the lesion, thereby reducing the likelihood of positive margins and subsequent recurrence. Additionally, it emphasizes the importance of rigorous postoperative monitoring, especially for patients with positive margins, to manage and mitigate the risk of HSIL recurrence effectively.

In addressing the treatment of HSIL through conization, it is also crucial to consider the long-term reproductive consequences associated with different surgical techniques. While more extensive tissue removal can decrease the risk of positive resection margins and reduce the recurrence of CIN2 + , it is associated with increased risks in subsequent pregnancies. Research, including findings by Kyrgiou [9] and a meta-analysis by Arbyn [8], has demonstrated that treatments like CKC significantly elevate the risks of perinatal mortality, severe and extreme preterm delivery, and the birth of low weight infants under 2000 g. Similarly, Liu [10] has documented that the Loop Electrosurgical Excision Procedure (LEEP) is linked with a higher incidence of preterm delivery, premature rupture of fetal membranes, and low birth weight infants. These adverse outcomes highlight the importance of surgical prudence and the need for individualized treatment planning that considers both oncologic safety and future pregnancy outcomes. Hence, while strategizing treatments for HSIL, especially in younger women planning future pregnancies, a careful evaluation of the extent of tissue removal is imperative to ensure optimal long-term health outcomes.

In our study, the positive rate of surgical margins was 12.9%. HPV16/18 infection, multiple HR-HPV infection, glandular involvement, Lesion quadrant ≥ 3 quadrants, TCT ≥ ASC-H and TZ III were independent risk factors for positive surgical margins after conization, which was consistent with literature reports [11, 12]. The potential risk factors leading to a positive margin may be consistent in CKC and LEEP, as well as the advantages of LEEP in mitigating bleeding, shortening recovery time, cervical stenosis and cervical incompetence, we believe the findings from this study, which are based on women treated with CKC, could also be applicable to populations treated with LEEP.

### HR-HPV persistent infection is an important cause of cervical precancerous

lesions and cervical cancer, among which HPV16/18 is the most closely related, and multiple HR-HPV infections can also increase the risk [13, 14]. In this study, the infection rate of HR-HPV was 88.4%, and the infection rate of HPV16/18 was 51.6%. Therefore, HR-HPV detection is extremely important in cervical cancer screening. The long-term presence of HPV in cervical epithelial cells can lead to decreased immunity, persistent infection in the reproductive tract, accelerated cell proliferation, inhibited cell apoptosis, and disordered environmental regulation in the reproductive tract, thereby aggravating tissue infiltration and increasing the possibility of positive surgical margins after surgery [15]. Kang [16] analyzed the relationship between HR-HPV infection types and cervical lesions, and believed that positive surgical margins after cervical conization were closely related to persistent infection of various types of HR-HPV in patients. In this study, we found that HPV16/18 and multiple HR-HPV infections were both independent risk factors for positive surgical margins. Therefore, HR-HPV detection plays an important role in cervical cancer screening and follow-up after HSIL treatment. Similarly, TCT results are also important risk factors for positive resection margins. A retrospective study found that the preoperative TCT results of the positive resection margin group were mainly HSIL and ASC-H, and the negative group were mainly NILM and ASC-US [17]. In this study, there were 432 patients with positive margins, of which 283 patients had severe cytological abnormalities (HSIL and ASC-H) before surgery. Among 2911 patients with negative margins, 466 (16%) had severe cytological abnormalities, and 2274 (84%) had no or mild cytological abnormalities. There is some controversy about the association between glandular involvement and positive surgical margins [12, 17–19]. In this study, glandular involvement was the risk factor, The reason for positive surgical margins caused by recurrent glands may be that the normal columnar cells located in the cervical glands are replaced by atypical cells. However, due to the deep lesion, these atypical cells are covered by normal epithelium, resulting in no positive results of cytology and colposcopy, which results in positive surgical margins after surgical removal of the covered epithelial tissue [20]. Regarding lesion quadrant, a cross-sectional study showed that HSIL lesions more than 2 quadrants were a high risk factor for positive margins and residual lesions after conization [21], which is consistent with literature reports. In addition to the above factors, the TZ III is also a high risk factor for positive surgical margins after CKC. This may be due to the displacement of the cervical squamous transformation zone, the deep position of the lesion, and the inability to completely remove the lesion tissue. Wang [18] showed that 61.08% of the patients in the positive margin group had TZ III, while only 38.92% of the patients in the negative margin group had TZ III. The risk of positive resection margin in the TZ III was about 2.99 times higher than that in the TZ I/II. Therefore, patients with high risk factors should strengthen individualized management and strict follow-up after surgery.

Based on the preoperative exposure factors and detection indicators collected in patients with HSIL, this study constructed a positive risk prediction model for postoperative margin of CKC. On the one hand, based on the comprehensive comparison of the prediction accuracy, sensitivity, specificity, PPV, NPV and AUC results of various prediction models in the test set, LR prediction model has better prediction effect and can be widely used in clinical prediction of the risk of positive surgical margin after CKC surgery. On the other hand, the parameter variables involved in the model can be obtained through simple medical history inquiry and routine clinical testing, without complex operations or invasive and expensive examinations, and without any privacy issues, so the model is popular and acceptable. In practical clinical application, the model can be compiled as a program and saved in the computer. After the clinician enters the corresponding values of relevant predictive variables into the program according to the patient's medical history data, the computer will automatically calculate the risk of positive margins after CKC surgery, so that the high-risk patients with positive margins after CKC surgery can be quickly and effectively identified before surgery. For patients identified as high-risk before surgery, clinicians should give timely and adequate evaluation before surgery and formulate individualized diagnosis and treatment plans, such as selection of conization scope or conization mode. At the same time, high-risk groups should be closely followed up after conization, so as to reduce the risk of residual and recurrence during follow-up.

This study still has some limitations. Firstly, this is only a retrospective study and selection bias is inevitable. Second, this study only focused on the effect of HPV16/18 infection on positive surgical margins and ignored the effect of other HR-HPV infections. Third, the postoperative pathological examination basically took HSIL as the diagnostic report and did not distinguish between CIN 2 and CIN 3, Fourth, Both the predictive model construction cohort and the external validation cohort were CKC patients from one hospital, and the models could not be used in other hospitals due to different conization methods and conization ranges.

In conclusion, our study identifies several independent risk factors for positive resection margins following CKC, including HPV16/18 infection, multiple HR-HPV infections, glandular involvement, lesions covering three or more quadrants, TCT results of at least ASC-H, and involvement of the TZ III. The clinical prediction model developed from these findings demonstrates robust consistency and practical value, offering significant guidance for clinicians in managing and following up with patients postoperatively. This model not only aids in surgical planning but also enhances post-treatment monitoring, ultimately contributing to improved patient outcomes.

## Data Availability

The datasets generated and analysed during the current study are not publicly available due avoid unreasonable use by third parties or organizations, but are available from the corresponding author on reasonable request.

## Abbreviations

CKC: Cold knife conization
HSIL: High-grade squamous intraepithelial lesion
CAP: American Society for Pathology
ASCCP: American Society for Colposcopy and Cervical Pathology
CIN: Cervical intraepithelial neoplasia
HR-HPV: High-risk human papillomavirus
TCT: Thincytologic test
ROC: Receiver operator characteristic
AUC: Area under receiver operator characteristic curve
DCA: Decision curve analysis
NILM: Negative for intraepithelial lesion or malignancy
LSIL: Low grade cervical intraepithelial lesions
HSIL: High grade cervical intraepithelial lesions
ASCUS: Atypical squamous cells of undetermined significance
ASC-H: Atypical squamous cells, cannot exclude high-grade squamous intraepithelial lesion
TZ: Transformation zone of the cervix
LEEP: Loop Electrosurgical Excision Procudure
